# IL-32γ promotes the healing of murine cutaneous lesions caused by *Leishmania braziliensis* infection in contrast to *Leishmania amazonensis*

**DOI:** 10.1186/s13071-017-2268-4

**Published:** 2017-07-14

**Authors:** Rodrigo Saar Gomes, Muriel Vilela Teodoro Silva, Jéssica Cristina dos Santos, Lucas Luiz de Lima Silva, Aline Carvalho Batista, Juliana Reis Machado, Mauro Martins Teixeira, Miriam Leandro Dorta, Milton Adriano Pelli de Oliveira, Charles A Dinarello, Leo A. B. Joosten, Fátima Ribeiro-Dias

**Affiliations:** 1 Instituto de Patologia Tropical e Saúde Pública, Universidade Federal de Goiás, Goiânia, Goiás Brazil; 2 Faculdade de Odontologia, Universidade Federal de Goiás, Goiânia, Goiás Brazil; 3 Departamento de Bioquímica e Imunologia, Universidade Federal de Minas Gerais, Belo Horizonte, Minas Gerais Brazil; 4 Division of Infectious Diseases, School of Medicine, University of Colorado Denver, Aurora, CO USA; 5 Department of Internal Medicine and Radboud Center of Infectious Diseases (RCI), Radboud University Medical Center, Nijmegen, The Netherlands

**Keywords:** *Leishmania amazonensis*, *Leishmania braziliensis*, Cutaneous leishmaniasis, IL-32, Cytokines, Mouse model

## Abstract

**Background:**

Interleukin 32 (IL-32) is a pro-inflammatory cytokine induced in patients with American tegumentary leishmaniasis (ATL) caused by *Leishmania braziliensis*. Here, we investigated whether IL-32 is also expressed in patient lesions caused by *L. amazonensis.* In addition, we evaluated experimental *L. amazonensis* and *L. braziliensis* infections in C57BL/6 transgenic mice for human IL-32γ (IL-32γTg) in comparison with wild-type (WT) mice that do not express the IL-32 gene.

**Results:**

Human cutaneous lesions caused by *L. amazonensis* express higher levels of IL-32 than healthy control skin. In mice, the presence of IL-32γ promoted the control of cutaneous lesions caused by *L. braziliensis*, but not lesions caused by *L. amazonensis* in an ear dermis infection model. In addition, IL-32γTg mice displayed less tissue parasitism and inflammation in IL-32γTg than WT mice during the healing phase of *L. braziliensis* infection. Production of antigen-specific pro-inflammatory cytokines was higher in IL-32γTg mice than in WT mice during *L. braziliensis* infection but not during *L. amazonensis* infection.

**Conclusions:**

Human cutaneous lesions caused by *L. amazonensis* express high levels of IL-32*.* In mice, the presence of IL-32γ contributes to the lesion healing caused by *L. braziliensis* but not by *L. amazonensis*. Data suggest that despite the ability for both species to induce IL-32 in humans, the connections between this cytokine and other immune players induced by related species of parasites can lead to distinct outcomes of the murine infections.

## Background

American tegumentary leishmaniasis (ATL) is an infectious disease caused by *Leishmania* protozoan, affecting the skin, oral or nasal mucosa. Brazil is one of 10 countries that together account for 70–75% of the cases of tegumentary leishmaniasis in the world [1]. *Leishmania amazonensis* and *Leishmania braziliensis* are the main species that cause ATL in Brazil. *L. amazonensis* is associated with the development of localized or diffuse skin lesions whereas *L. braziliensis* is associated with localized cutaneous or mucosal lesions [2–4].

In general, humans or mice infected with *L. braziliensis* present a stronger cellular immune response against the parasites than human or mice infected with *L. amazonensis* [5, 6]. *L. braziliensis* infections cause small cutaneous lesions that regress after a few weeks in C57BL/6 mice. In these mice, it has been demonstrated that the IL-12-IFN-γ/TNF-α-NO axis controls the parasite infection [7–9]. By contrast, *L. amazonensis* generates chronic and non-healing infection in C57BL/6 mice with a deficient Th1 cell response [5, 10].

IL-32 is a cytokine expressed by several human cells, including NK cells, monocytes/macrophages, T lymphocytes, epithelial cells, endothelial cells, fibroblasts and hepatocytes. IL-32 is predominantly expressed intracellularly and can induce the production of TNF-α, IL-8 and IL-1β [11]. To date, there are nine isoforms of human IL-32 and the highest biological activity has been attributed to IL-32γ [12]. IL-32 is associated with the control or immunopathology of numerous infectious diseases, such as tuberculosis, HIV/AIDS, leprosy and hepatitis [13] likewise in dermatological diseases [14, 15].

Although rodents do not naturally produce IL-32, recombinant IL-32 (rIL-32) can activate mouse cells [11]. In addition, injection of rIL-32γ into the knee joints of mice leads to arthritis partially mediated by induction of TNF-α [16]. Thus, the use of an experimental animal model to study the role of IL-32 in inflammatory and infectious diseases is made possible by IL-32 humanized transgenic mice.

We previously described that cutaneous and mucosal lesions of patients with ATL caused by *L. braziliensis* exhibit increased IL-32 expression compared to healthy tissues [17]. Here, we investigated the expression of IL-32 in cutaneous lesions of patients infected with *L. amazonensis*, and the role of IL-32γ in experimental mouse infections caused by *L. amazonensis* and *L. braziliensis*.

## Methods

### Patient and control samples

Patients diagnosed with cutaneous leishmaniasis according to Oliveira et al. [8] and control healthy individuals were submitted to biopsy procedure to obtain fragments from lesions and healthy skin, after signing the consent form.

### Immunohistochemical (IHC) analysis for IL-32

Biopsy fragments were obtained from cutaneous lesions to identify *Leishmania* species by polymerase chain reaction (PCR) as previously described [8], confirming all samples positive for *L. amazonensis*. Samples of healthy skin (*n* = 8) and a fragment of lesions from patients (*n* = 5) were used for IHC analysis for IL-32 using rabbit polyclonal antibodies to human IL-32 (Abcam Inc., Cambridge, UK), according to Galdino et al. [17]. The tissue expression (epithelium and dermis) of IL-32 was classified as follows: 0, absence of labelled cells; 1, 1–25% of labelled cells; 2, 26–50% of labelled cells; 3, 51–75% of labelled cells; and 4, 76–100% of labelled cells). All sections were blindly analyzed using a light microscope (magnification of 400×).

### Animals and parasites

Transgenic mice for human IL-32 were developed by Choi et al. [18] and donated to our group by Dr. Charles Dinarello (University of Colorado, Denver, USA). Six to 8 week old C57BL/6 WT and IL-32γTg mice were used in the experiments. All procedures were followed in accordance with the guidelines and legislation on ethics research.

*Leishmania* (*L.*) *amazonensis* (MHOM/BR/1973/M2269) and *L.* (*V.*) *braziliensis* (MHOM/BR/2003/IMG) strains were obtained from patients with localized cutaneous lesions [19, 20]. *L. braziliensis* strain was obtained and identified by our group, as described by Dorta et al. [19]. Briefly, lesion fragments were macerated in phosphate-buffered saline (PBS) and cultured in Grace’s Insect Medium (Gibco, Life Technologies, Carlsbad, USA) supplemented with heat-inactivated 20% fetal bovine serum (FBS, Sigma-Aldrich, St. Louis, USA) and 100 U/ml of penicillin/streptomycin (Sigma-Aldrich) at 26 °C. The identification of the species was performed by PCR-RFLP, according to Volpini et al. [21]. *Leishmania amazonensis* strain was identified and donated to us by Mortara et al. [20].

*Leishmania amazonensis* and *L. braziliensis* promastigote forms were cultured in Grace’s insect medium supplemented as described above. Parasites of either *L. amazonensis* or *L. braziliensis* from stationary phase (6th day) of growth were washed three times with sterile PBS, pH 7.4 (1000×*g*, 10 min, 10 °C), suspended in PBS and quantified by hemocytometer after fixation with PBS/0.1% formaldehyde.

Parasite lysates were obtained by 5 freeze-thaw cycles in liquid nitrogen and 37 °C water bath followed by protein quantification using the Pierce BCA protein assay (ThermoFisher, Rochester, USA).

### Infection, disease progression and histopathological analysis

Animals were inoculated (1 × 10^5^ promastigotes/10 μl of PBS) into the dermis of the left ear. Three independent experiments were performed, with three animals per group in each experiment. Lesion size was measured weekly using a digital caliper. Lesion size is described as the difference between the thickness of the infected ear and the thickness of the uninfected ear [22]. Tissue parasitism was evaluated in the infected ear, draining lymph node (submandibular) and spleen by limiting dilution assay. The results were expressed as the negative logarithm of the parasite titer [23]. Paraformaldehyde fixed-ear tissue was embedded in paraffin to be processed for histopathological analysis after haematoxylin and eosin (H&E) staining. The cellular infiltrate evidenced in the inflammatory process was considered [24–26].

### Cytokine production

Lesion-draining submandibular lymph node cells from uninfected and infected mice were macerated and maintained in RMPI 1640 medium (Sigma-Aldrich) supplemented with 10% FBS (Gibco), 1 M HEPES (Sigma-Aldrich), 2 mM glutamine (Sigma-Aldrich), 100 U/ml penicillin (Sigma-Aldrich) and 100 μg/ml streptomycin (Sigma-Aldrich). Viable cells were quantified using a hemocytometer by dye exclusion with Trypan blue 0.1% in PBS. Lymph node cells (5 × 10^5^ cells/ml) were stimulated with antigen from *L. amazonensis* or *L. braziliensis* (50 μg/ml) for 24 h or 72 h, at 37 °C and 5% CO_2_. TNF-α and IL-10 were evaluated in the culture supernatants by commercial enzyme-linked immunosorbent assay (ELISA) kits (R&D Systems, Minneapolis, USA), according to the manufacturer’s protocol. IFN-γ was evaluated by ELISA using monoclonal antibodies obtained from hybridoma cultures, according to [27]. All cultures and measurements were done in duplicates.

### Statistical analysis

Data are expressed as means ± standard deviations or median and individual values and compared using Student’s *t* or Mann-Whitney U tests, respectively. Analyses were performed using Prism software version 6.0 (GraphPad, San Diego, CA, USA). Significance was established as *P* < 0.05.

## Results

### Expression of IL-32 in lesions of patients with ATL caused by *L. amazonensis*

**Fig. 1:**
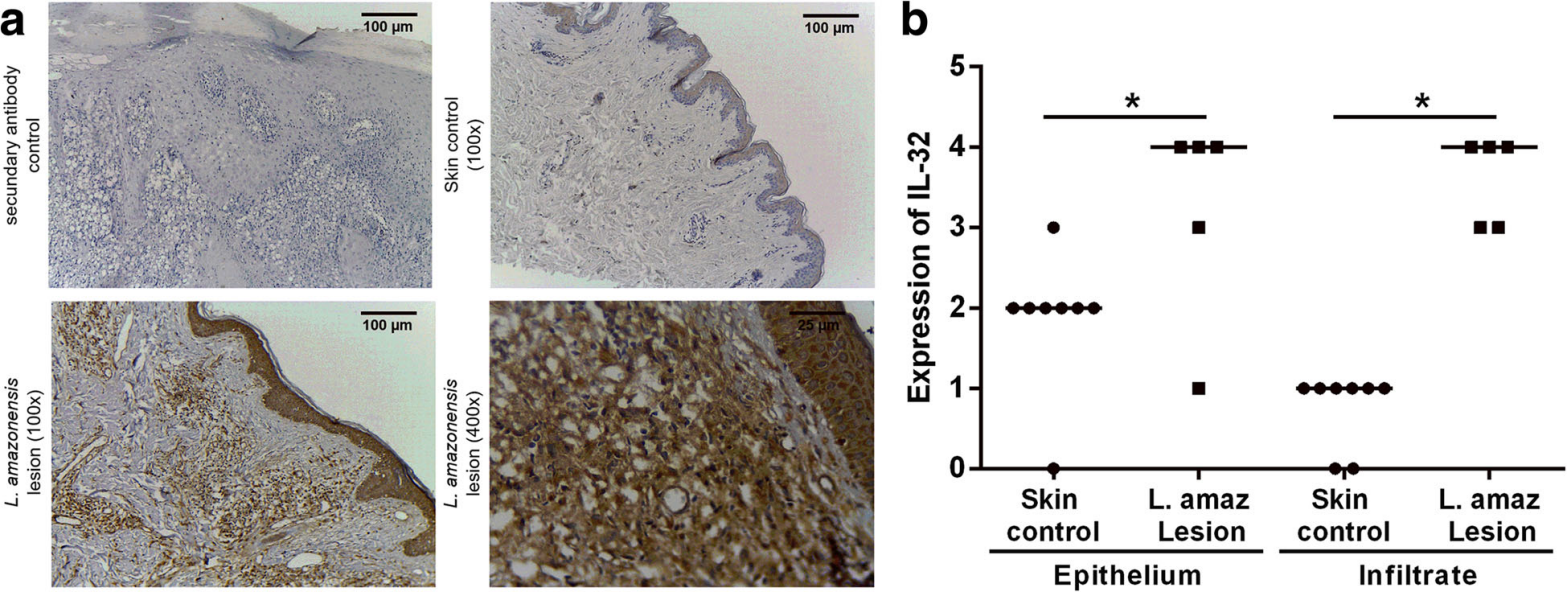
Expression of IL-32 in American tegumentary leishmaniasis lesions caused by *L. amazonensis*. **a** Fragments of lesions from ATL patients infected with *L. amazonensis* and skin from healthy controls were included in paraffin and submitted to immunohistochemistry for IL-32. The reaction was revealed with 3,3′-Diaminobenzidine and Meyer’s hematoxylin used to counterstain. **b** Evaluation of IL-32 expression score was determined according to the percentage of cells expressing IL-32. The scores represent: 0 (absence of stained cells), 1 (1–25% of stained cells), 2 (26–50% stained cells), 3 (51–75% stained cells), and 4 (76–100% stained cells). *P < 0.05 (Mann-Whitney test)

We previously demonstrated that IL-32 is highly expressed in cutaneous and mucosal lesions of patients with ATL infected with *Leishmania* (*Viannia*) spp., mainly *L. braziliensis*. In addition, amastigote forms of *L. braziliensis* were able to induce IL-32β in human peripheral blood mononuclear cells [17]. Here, we demonstrate that in cutaneous lesions caused by *L. amazonensis* infection IL-32 protein expression was also increased when compared to healthy skin specimens [*U* = 7.5, *P* = 0.0412 (epithelium) and *U* = 0.0, *P* = 0.0008 (inflammatory infiltrate)]. IL-32 was detected both in the epithelium and in the inflammatory infiltrate (Fig. 1a, b).

### The role of IL-32 in experimental lesions caused by *L. amazonensis* or *L. braziliensis*

**Fig. 2:**
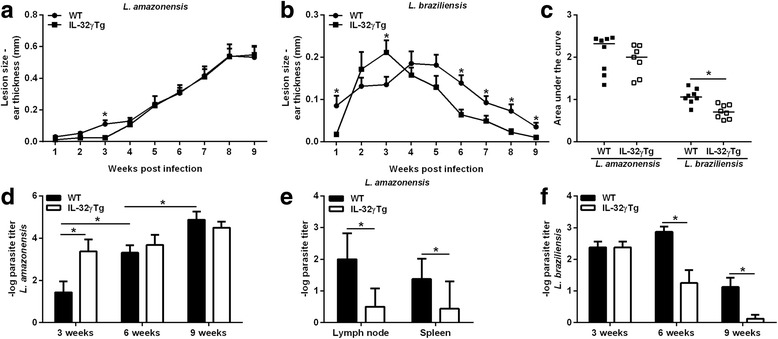
IL-32 promotes the control of cutaneous infection caused by *L. braziliensis*, but not caused by *L. amazonensis* in an ear dermis model. WT and IL-32γTg mice were infected in the left ear with 1 × 10^5^ *L. amazonensis* (IFLA/BR/67/PH8) or *L. braziliensis* (MHOM/BR/2003/IMG) promastigotes in the stationary phase of growth (6 days of culture). **a** Ear thickness was monitored weekly, and lesion size was determined by the difference between infected and uninfected ears. **a** *L. amazonensis* infection. **b** *L. braziliensis* infection. **c** Area under the curves is shown for each animal. **d** Ear parasite numbers were determined on week 3, 6 and 9 post-infection with *L. amazonensis* using limiting dilution assays. **e** Lymph node and spleen parasite numbers were determined on week 9 post-infection with *L. amazonensis.* **f** Ear parasite numbers were determined on week 3, 6 and 9 post-infection with *L. braziliensis* using limiting dilution assays. The results presented mean ± standard deviation (8 animals per group). *P < 0.05 (WT × IL-32γTg, Student’s *t*-test)

To understand the precise role of IL-32 in ATL, we infected human IL-32γTg and WT mice with either *L. amazonensis* or *L. braziliensis*. IL-32γTg mice showed a delayed development of ear lesions caused by *L. amazonensis* (until week 3); however, the size of the lesions were similar to WT mice in later stages of infection (Fig. 2a, c). In contrast, mice infected with *L. braziliensis* demonstrated a significant increase in the lesion size on the 3rd week of infection (*t*_(14)_ = 2.23316, *P* = 0.042), as well as a reduction of lesion size from week 6 post-infection in IL-32γTg mice compared to WT mice (Fig. 2b, c) [*t*_(14)_ = 3.29151, *P* = 0.0053 (6 weeks); *t*_(14)_ = 2.16645, *P* = 0.048 (7 weeks); *t*_14)_ = 2.82843, *P* = 0.013 (8 weeks); *t*_(14)_ = 2.37595, *P* = 0.032 (9 weeks)].

**Fig. 3:**
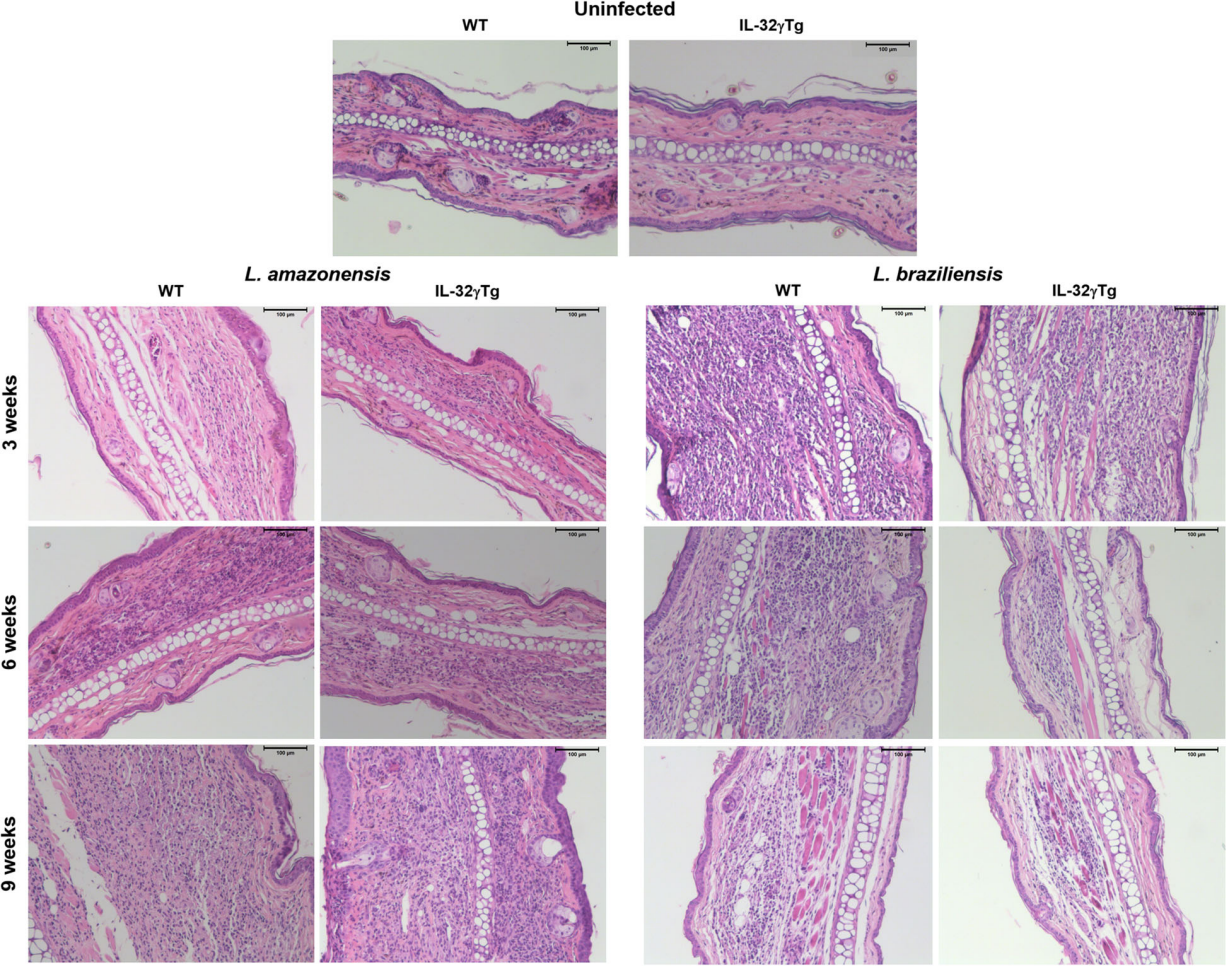
Histopathological profiles of ear lesions caused by *L. amazonensis* and *L. braziliensis* in wild-type and IL-32γTg mice. Fragments of ears from C57BL/6 WT and IL-32γTg mice uninfected (above) or infected with *L. amazonensis* (left) or *L. braziliensis* (right) for 3, 6 and 9 weeks were fixed and stained by hematoxylin & eosin, for analysis under a light microscope (100× magnification). A mononuclear cell inflammatory infiltrate was observed in the dermis that was progressive in *L. amazonensis*-infected and regressive in *L. braziliensis*-infected mice. *Scale-bars*: 100 μm

We observed that *L. amazonensis*-infected IL-32γTg mice showed higher lesional parasite load on week 3 of infection compared to WT animals (*t*_(14)_ = 2.52036, *P* = 0.024). Despite this initial favoring of parasitism in IL-32γTg animals, the growth of *L. amazonensis* in later stages of infection was controlled, whereas parasites grew exponentially in WT mice (Fig. 2d). Although IL-32γ was not able to reduce the *L. amazonensis* infection in the skin, we observed that IL-32γ is important for parasite dissemination because IL-32γTg mice harbored lower numbers of *L. amazonensis* parasites in draining lymph nodes (*t*_(6)_ = 3, *P* = 0.024) and spleens (*t*_(14)_ = 2.46598, *P* = 0.027) compared with WT mice in 9 weeks (Fig. 2e). During *L. braziliensis* infection, we observed a strong reduction of parasite burden in IL-32γTg mice [*t*_(14)_ = 3.68751, *P* = 0.0024 (week 6) and *t*_(14)_ = 3.12076, *P* = 0.0075 (week 9)] (Fig. 2f). We did not observe *L. braziliensis* dissemination until the end of the experiments. As expected, WT and IL-32γTg mice exhibited similar histological profiles of lesional inflammatory infiltrates after *L. amazonensis* infection. In contrast, the inflammatory infiltrate was remarkably reduced on weeks 6 and 9 post-infection in *L. braziliensis*-infected IL-32γTg mice compared to WT mice. Despite this, both WT and IL-32γTg mice showed a reduction of inflammatory infiltrate at the final stage of infection (9 weeks) when comparing the histopathological aspects on 3 or 6 weeks (Fig. 3).

### IL-32γ induces inflammatory cytokines in experimental infections caused by *L. braziliensis* in contrast to *L. amazonensis*

**Fig. 4:**
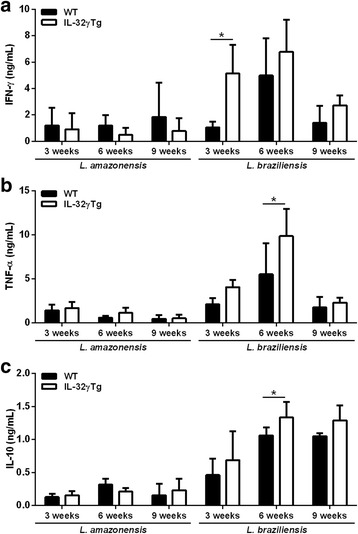
IL-32 amplifies the production of inflammatory cytokines induced by *L. braziliensis* during murine ear dermis infection. Lesion-draining submandibular lymph node cells were stimulated with specific antigens from *L. amazonensis* or *L. braziliensis* for 24 h (TNF-α) or 72 h (IFN-γ and IL-10). The production of (**a**) IFN-γ, (**b**) TNF-α and (**c**) IL-10 was evaluated by ELISA in culture supernatants. The results represent the mean ± standard deviation of 8 animals per group. *P < 0.05, (WT × IL-32γTg, Student’s t-test)

Lesion-draining submandibular lymph node cells from infected mice were stimulated ex vivo with lysates of *L. amazonensis* or *L. braziliensis* promastigotes for 24 h, or 72 h and cytokines were measured in culture supernatants. No difference in the production of IFN-γ, TNF-α and IL-10 was observed between WT and IL-32γTg animals infected with *L. amazonensis* on weeks 3, 6 or 9 post-infection. In contrast, lymph node cells from *L. braziliensis*-infected IL-32γTg animals showed higher cytokine production after antigen-specific stimulation than cells from WT mice (weeks 3 and 6; Fig. 4) [*t*_(51)_ = 3.22153, *P* = 0.0022 (IFN-γ, 3 weeks); *t*_(54)_ = 4.87909, *P* < 0.0001 (TNF-α, 6 weeks); *t*_(14)_ = 2.92794, *P* = 0.011 (IL-10, 6 weeks)].

## Discussion

The present study demonstrated that in addition to *L. braziliensis* [17], *L. amazonensis* induces a strong IL-32 expression in human cutaneous lesions. Furthermore, we showed that human IL-32γ was able to support the healing of skin lesions in *L. braziliensis*-infected mice, which is in contrast to *L. amazonensis* caused skin lesions. Based on pro-inflammatory properties of IL-32γ [11, 13] we hypothesize that IL-32γ could increase the immune response against *L. amazonensis* and lead to improved healing of the lesions. Although IL-32γ inhibited the lesional parasite load, IL-32γ did not increase the production of pro-inflammatory cytokines or improved healing. It is well-known that *L. amazonensis* strongly modulates the host immune response against this parasite [5, 10, 18, 28]. These experimental results, together with the observation that IL-32 is highly expressed in cutaneous lesions of *L. amazonensis*-infected patients, suggest that even in the presence of IL-32 the parasites and lesion are persistent.

Although it is known that C57BL/6 mice are relatively resistant to *L. braziliensis* infection [5], we observed that the pro-inflammatory response during *L. braziliensis* infection was strengthened by IL-32γ without a significant increase of immunopathology. In addition, IL-32γ played an important role in controlling the parasite load, favoring the healing of the skin lesion. Thus, in cutaneous murine lesions caused by *L. braziliensis* that is restricted to skin and draining lymph node, the pro-inflammatory properties of IL-32γ help the control of the infection. However, in *L. amazonensis,* which spreads beyond the cutaneous lesions to other tissues and suppress cytokine production, IL-32γ contributes to the control of parasite dissemination but not for skin lesion healing. Differential expression of IL-32γ in the tissues during these two infections could explain the results besides intrinsic parasite factors that can interfere with the role of IL-32γ. The results underscore the need of unravelling the molecular mechanisms used by *L. amazonensis* parasites to subvert the antileishmanial effect of IL-32γ in skin observed against *L. braziliensis* infection.

It is important to highlight that murine models of leishmaniasis are not a reliable landscape of the immune responses against *Leishmania* parasites because mice do not produce IL-32. However, murine cells respond to human IL-32 [12]. Some important microbicidal mechanisms are dependent on IL-32, and therefore these latter mechanisms are lost in mice [29]. In this way, this IL-32γTg mouse model is very important to reveal novel mechanisms that control or lead to immunopathogenesis in leishmaniasis.

## Conclusions

IL-32γ is an important player in the control of *L. braziliensis* cutaneous infections in contrast to *L. amazonensis-*mediated infections, at least in ear dermis infection model. IL-32γ might be a novel target in strategies to control leishmaniasis caused by *L. braziliensis*.

## Abbreviations

ATL: American tegumentary leishmaniasis
IFN-γ: Interferon gamma
IL-10: Interleukin-10
IL-12: Interleukin-12
IL-1β: Interleukin 1 beta
IL-32: Interleukin-32
IL-8: Interleukin-8
NK: Natural killer cell
NO: Nitric oxide
Th1: Type 1 T helper cells
TNF-α: Tumor necrosis factor-alpha
WT: Wild-type
